# Identification and Annotation of Potential Function of Regulatory Antisense Long Non-Coding RNAs Related to Feed Efficiency in *Bos taurus* Bulls

**DOI:** 10.3390/ijms21093292

**Published:** 2020-05-06

**Authors:** Wietje Nolte, Rosemarie Weikard, Ronald M. Brunner, Elke Albrecht, Harald M. Hammon, Antonio Reverter, Christa Kühn

**Affiliations:** 1 Institute of Genome Biology, Leibniz Institute for Farm Animal Biology (FBN), 18196 Dummerstorf, Germany; nolte@fbn-dummerstorf.de (W.N.); weikard@fbn-dummerstorf.de (R.W.); brunner@fbn-dummerstorf.de (R.M.B.); 2 Institute of Muscle Biology and Growth, Leibniz Institute for Farm Animal Biology (FBN), 18196 Dummerstorf, Germany; albrecht@fbn-dummerstorf.de; 3 Institute of Nutritional Physiology “Oskar Kellner”, Leibniz Institute for Farm Animal Biology (FBN), 18196 Dummerstorf, Germany; hammon@fbn-dummerstorf.de; 4 Commonwealth Scientific and Industrial Research Organisation (CSIRO) Agriculture and Food, Queensland Bioscience Precinct, St Lucia 4067 QLD, Australia; toni.reverter-gomez@csiro.au; 5 Faculty of Agricultural and Environmental Sciences, University Rostock, 18059 Rostock, Germany

**Keywords:** *Bos taurus*, feed efficiency, co-expression network analysis, lncRNA, Functional Annotation of Animal Genomes (FAANG)

## Abstract

Long non-coding RNAs (lncRNAs) can influence transcriptional and translational processes in mammalian cells and are associated with various developmental, physiological and phenotypic conditions. However, they remain poorly understood and annotated in livestock species. We combined phenotypic, metabolomics and liver transcriptomic data of bulls divergent for residual feed intake (RFI) and fat accretion. Based on a project-specific transcriptome annotation for the bovine reference genome ARS-UCD.1.2 and multiple-tissue total RNA sequencing data, we predicted 3590 loci to be lncRNAs. To identify lncRNAs with potential regulatory influence on phenotype and gene expression, we applied the regulatory impact factor algorithm on a functionally prioritized set of loci (*n* = 4666). Applying the algorithm of partial correlation and information theory, significant and independent pairwise correlations were calculated and co-expression networks were established, including plasma metabolites correlated with lncRNAs. The network hub lncRNAs were assessed for potential *cis*-actions and subjected to biological pathway enrichment analyses. Our results reveal a prevalence of antisense lncRNAs positively correlated with adjacent protein-coding genes and suggest their participation in mitochondrial function, acute phase response signalling, TCA-cycle, fatty acid β-oxidation and presumably gluconeogenesis. These antisense lncRNAs indicate a stabilizing function for their *cis*-correlated genes and a putative regulatory role in gene expression.

## 1. Introduction

While the functionality of protein-coding genes has been thoroughly explored and scrutinized in the past century—and continues to be—the so-called ‘dark matter of the genome’ has shifted into focus in the recent decades. Non-coding elements are estimated to cover about 98% of the mammalian genome and to comprise different elements such as microRNAs, small nuclear RNAs, small nucleolar RNAs, transfer RNAs (miRNA, snRNA, snoRNA, tRNA, Encode Project Consortium [1]), the previously discovered circular RNAs (circRNA [2]), as well as long non-coding RNAs (lncRNAs). 

In cattle breeding and production, the efficient use of feed by the animal is continuously gaining importance for ecological and economic reasons. In the beef industry, residual feed intake (RFI) as a measure of feed efficiency is usually the measure of choice [3]. Numerous association studies have aimed to find causative genomic regions and gene variants that drive bovine feed efficiency, but repeatedly quantitative trait locus (QTL) peaks fall outside the protein-coding genes, e.g., [4,5,6,7]. This plethora of work suggests that the functional tasks of the non-coding elements affecting feed efficiency need to be addressed in greater detail. 

To date, functional annotation of lncRNAs is still not fully comprehensive in human and model animals and even less so in livestock species, although first advances are in progress. Known modes of action of lncRNAs include chromatin-remodelling and chromatin state maintenance, and transcriptional enhancement or repression, e.g., through the binding to transcriptional regulatory factors as reviewed by Long et al. [8] and Marchese et al. [9]. 

Increasing evidence has shown that lncRNAs are involved in a broad range of pathological and disease conditions and environmental transitions but also in the general regulation of immune and metabolic processes in normal cell and tissue homeostasis, e.g., by acting as signal molecules that mark the regulation of developmental and physiological stages and gene expression. Lu et al. [10] summarized results indicating that lncRNAs are able to reprogram glucose and lipid metabolism in tumor cells by modulating key enzymes of glycolysis, oxidative phosphorylation and pentose phosphate as well as lipid synthesis and degradation pathways. A recent comprehensive overview of lncRNAs involved in lipid metabolism [11] elucidated lncRNAs that are potentially associated with hepatic lipid and glucose metabolism and related to metabolic disorders, such as obesity, cardiovascular diseases and hepatic steatosis. In murine liver, Yang et al. [12] found a lncRNA with a pivotal effect on lipogenesis, which was documented to act through a negative feedback loop relationship with a transcription factor coding gene (*SREBP1c*). Recently, Pradas-Juni et al. [13] identified a transcription factor MAFG-lncRNA (obesity-repressed lincIRS2) axis controlling hepatic glucose metabolism in health and metabolic disease.

LncRNAs are generally categorized as genic or intergenic RNA classes, which can be transcribed as sense- or antisense-oriented with respect to their nearest neighbouring protein-coding gene. Antisense lncRNAs, originating from the complementary strand of protein-encoding genes, comprise a major proportion of lncRNAs in the transcriptome across species, e.g., [14,15,16]. They commonly link neighbouring or overlapping genes in complex loci into chains of transcriptional units [15]. The genomic arrangement of antisense RNAs and opposite sense genes suggests that they might be part of self-regulating circuits that allow afflicted genes to regulate their own expression [16]. Antisense lncRNAs can act in *cis* as stabilizers [17], thereby increasing the abundance of the respective transcripts and protein of the protected gene [18]. Facilitated through the introduction of stranded library protocols in the 2000s, many genes have been found to overlap with antisense non-coding genomic elements, so-called natural antisense transcripts (NATs). 

Although there are examples of lncRNAs with high sequence conservation across mammals, e.g., *MALAT1* [19], there is also evidence for a high level of sequence species-specificity in this RNA class compared with protein-coding genes [20]. For this reason, the identification and characterization of phenotype-influencing lncRNAs in the respective target species and tissue are advisable and one of the declared goals of the global initiative for Functional Annotation of Animal Genomes (FAANG, www.animalgenome.org/community/FAANG/). 

While there are a variety of bioinformatics tools at hand for the prediction of long non-coding sequences from transcriptomic data (e.g., PLEK [21], FEELnc [22], PLAR [23], CPC [24], CPAT [25], and CNCI [26]), the functional annotation of novel non-coding loci remains challenging. Common practice is to construct co-expression networks and use correlation partners of lncRNAs for gene and pathway enrichment analyses. This guilt-by-association approach has been applied to non-coding elements, such as miRNAs [27,28,29] and lncRNAs [30,31,32,33]. 

In a previous study, we applied a combination of the regulatory impact factor (RIF [34]) and a partial correlation and information theory (PCIT [35]) to build correlation networks to predict key regulatory lncRNAs with an implication in metabolic efficiency in crossbred cattle [36]. We integrated phenotypic data, plasma metabolite profiles and transcriptomic data from four tissues (jejunum, liver, skeletal muscle, rumen) and two sexes. However, at that stage, little attention was given to the tissue-specificity of expression data [37] and likely molecular function of lncRNAs. Due to its central role in metabolic processes [38], the liver has repeatedly been the subject of transcriptomic studies, also with regard to bovine feed efficiency [39,40,41,42,43]. Therefore, in the present study, we adapted our analysis pipeline to the new bovine genome ARS-UCD.1.2 and to a single-tissue approach, where we aim to identify liver lncRNAs with high regulatory potential and a functional relation to feed efficiency. 

## 2. Results

### 2.1. Alignment and Mapping of RNA Sequencing Data

After quality trimming, the average sequencing depth of the RNA-sequencing libraries was 49.8 million read pairs per sample and the average alignment rate to the reference genome ARS-UCD.1.2 was 98.72% ± 0.26% (Table 1). The mapping of fragments to the project specific merged annotation (Supplement 1), which contained 30,806 loci and 82,628 transcripts after quality filtering, resulted in an average mapping rate of 85.98% ± 1.40%. 

### 2.2. Long Non-Coding RNA Prediction

The identification of lncRNAs with FEELnc based on the project-specific merged annotation and the bovine reference genome and annotation yielded a total of 6161 non-coding transcripts (3,590 loci) with a minimal length of 200 nt (results from FEELnc and differential expression analysis and information on structure and position for each transcript are listed in Supplement 2). 

Within the default window size (10,000 to 100,000 nt), a total of 19,184 interactions of 3,495 lncRNA loci (out of 3590 loci) with positional partner genes were predicted, while 95 lncRNA loci (corresponding to 202 of the 6161 lncRNA transcripts) remained without a potential positional interaction partner locus. Out of the 3,495 loci with a predicted interaction partner, 1799 lncRNAs were in the sense direction to the predicted partner gene and 1,696 lncRNAs were in the antisense direction to their partner gene. The majority of the lncRNAs with an interaction partner assigned (2955) were classified as genic, meaning that they overlapped with their predicted partner gene in the sense or antisense orientation, and 540 lncRNAs were intergenic. The overall average expression level of the 3590 lncRNA loci was 10.13 FPKM (± 325.21) with a median of 0.26 FPKM. 

In a locus-based approach, where we considered the transcript with the highest exon number for each lncRNA locus, we observed that strandedness was equally distributed among the 3590 loci (50.84% on the plus and 49.16% on the minus strand). The average number of exons per locus amounted to 4.52 ± 7.14 (media*n* = 3.00) and the geometric mean of the total exonic length was 1,723.78 nt.

### 2.3. Differential Metabolite Abundance 

**Figure 1:**
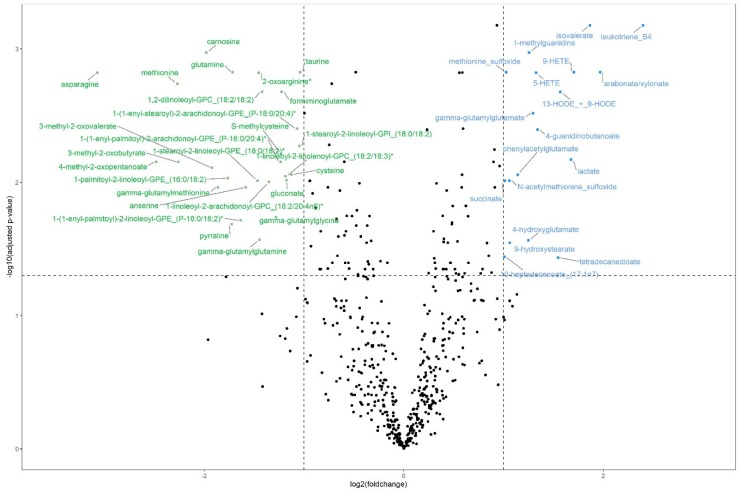
Volcano plot of differentially abundant plasma metabolites for bulls of high (*n* = 12) and low (*n* = 13) feed efficiency with upregulation (higher abundance) in highly efficient bulls with blue labels and downregulation (lower abundance) with green labels. Significance threshold (horizontal dotted line) at q (Benjamini-Hochberg) ≤ 0.05 and absolute log_2_(foldchange) ≥ 1 (vertical dotted lines).

Between the groups of high and low efficiency bulls, we found 45 plasma metabolites to be significantly differentially abundant (*q* (Benjamini–Hochberg) ≤ 0.05 and absolute log-transformed foldchange (log_2_FC) ≥ 1). Eighteen metabolites were downregulated, i.e., lower in abundance, in the high efficiency group and 27 were upregulated. The most pronounced differences were found for leukotriene B4 (*q* = 6.65 × 10^−4^; log_2_FC = 2.40) and isovalerate (*q* = 6.65 × 10^−4^; log_2_FC = 1.87), which were significantly higher in abundance in highly efficient bulls compared with the low efficiency group (see Figure 1, Supplement 3). The strongest downregulation in the high efficiency group was observed for asparagine (*q* = 1.51 × 10^−3^; log_2_FC = −3.07) and methionine (*q* = 1.83 × 10^−3^; log_2_FC = −2.27). Next to these two amino acids (AAs), the AAs glutamine and cysteine were also differentially abundant (*q* = 1.49 × 10^−3^; log_2_FC = −1.71 and *q* = 8.75 × 10^−3^; log_2_FC = −1.12, respectively).

**Figure 2:**
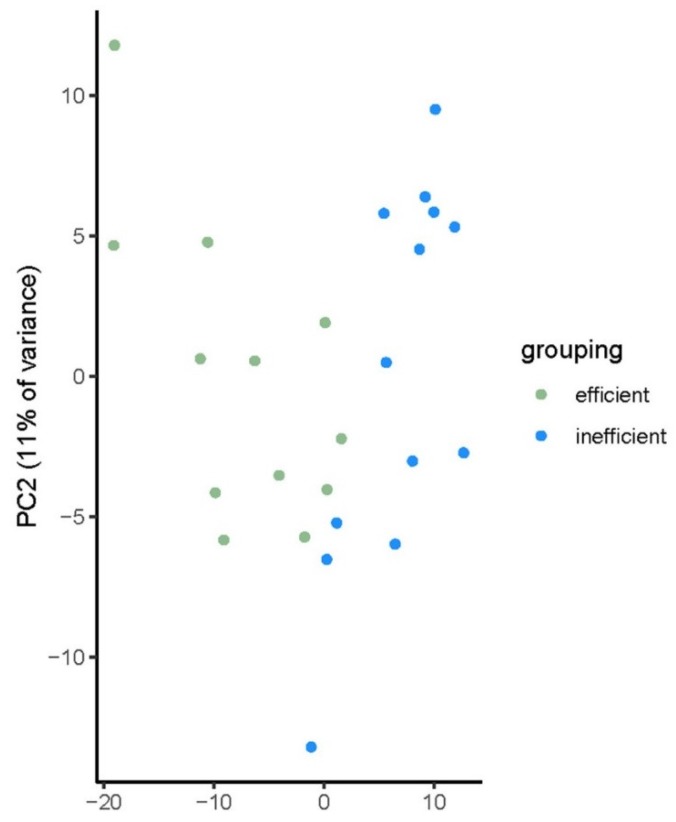
Principal component analysis (PCA) plot for 25 bulls divergent for feed efficiency. Plotting based on plasma metabolite levels (*n* = 552).

Plotting of a metabolite based principal component analysis (PCA) showed a clear separation of the bulls in the two efficiency groups (see Figure 2), with the first two components accounting for 38% of the variance in the metabolite abundance. 

### 2.4. Set of Prioritized Loci for Co-Expression Network

The AnimalQTL database listed 1573 QTL for RFI that stemmed from SNP array-based studies (manual curation of the complete dataset) and could be remapped to the new bovine reference genome ARS-UCD.1.2. Out of these 1573 QTLs, 1506 had a direct overlap with a locus in our project-specific merged annotation and no QTL was more than 3 Mb away from the next annotated locus. Finally, 843 of these loci passed the minimal expression threshold and were categorized as QTL locus. 

Out of the 745 loci that were significantly differentially expressed (DE) with *q* (Benjamini–Hochberg) ≤ 0.1 between the high and the low efficiency group, 219 were predicted to be lncRNAs (29.4%), and 84 out of the 843 QTL loci were also predicted to be lncRNAs (10.0%). 

**Figure 3:**
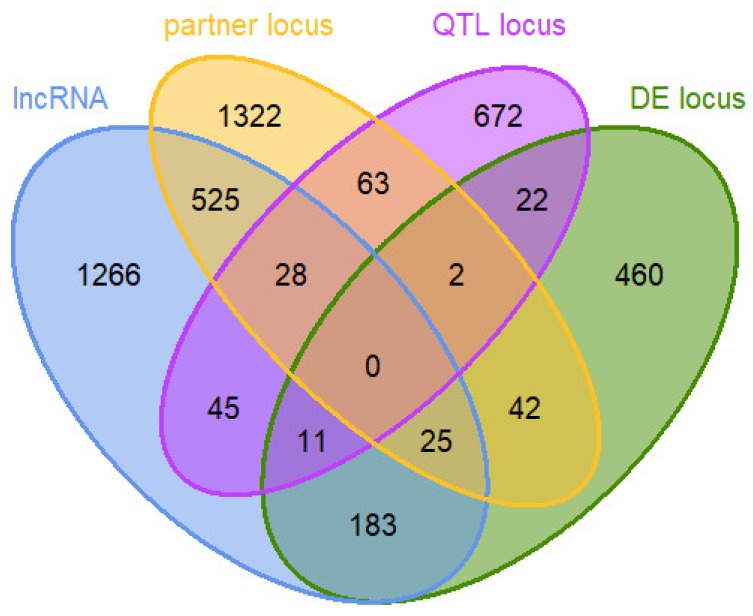
Venn diagram of 4666 loci in a prioritized loci set for co-expression network analysis: loci predicted to be lncRNAs (lncRNA) and their potential positional interaction gene partners (partner locus), loci overlapping with or no farther away than 3 Mb from a quantitative trait locus (QTL) for residual feed intake in cattle (QTL locus), and loci with differential expression (DE locus) between bulls of high and low feed efficiency.

In the end, the prioritized loci for the RIF and PCIT analyses contained a total of 4,666 unique loci, including 745 DE loci, 2083 lncRNAs, 2007 protein-coding partner gene loci, and 843 QTL loci (see Figure 3 and Supplement 4). Loci included in the prioritized loci set had to be minimally expressed (>0.1 FPKM in at least six animals of one efficiency group) and could fall into more than one category of the set. 

### 2.5. Regulatory Impact Factor Analysis

**Figure 4:**
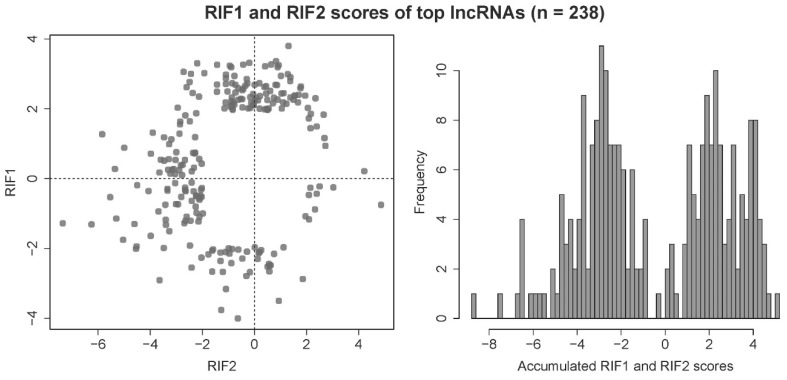
Distribution of scores of the metrics RIF1 and RIF2 from the regulatory impact factor (RIF) analysis for the top potential key regulatory lncRNAs, equalling 238 out of 2083 lncRNAs in the prioritized dataset (absolute z-transformed RIF1 or RIF2 ≥ 1.96).

Ultimately, 2083 lncRNAs and 3400 unique target loci (loci in the categories partner gene, QTL or DE locus) were submitted to the RIF analysis. In some cases, lncRNAs could be both potential regulators as well as target loci, hence the higher number of target loci. After z-transformation and filtering for lncRNAs with an absolute RIF1 or RIF2 score of ≥ 1.96, 238 lncRNAs were found to be significant and therefore potential key regulators in this dataset. As the two RIF metrics are designed to detect different mechanisms of regulation, the 238 key lncRNAs typically score either very high or very low in RIF1 or RIF2 and have a score around zero in the other metric, which results in a bimodal distribution of accumulated RIF scores (see Figure 4).

### 2.6. Co-Expression Networks Based on Partial Correlation and Information Theory Approach and Detection of Hub LncRNAs

The prioritized loci set (4666 loci) that was used for the RIF analysis was subsequently also submitted to the PCIT algorithm and results were filtered for significant pairwise correlations with a minimal strength of |r| ≥ 0.65, where one correlation partner had to be a lncRNA with a significant RIF score. This resulted in a total of 16,489 connections including 2299 out of the 4666 loci. After including significant (*p* ≤ 0.01) correlations between key lncRNAs and plasma metabolites (|r| ≥ 0.65) the co-expression networks comprised 2414 nodes with 16,709 edges. With 15,783 edges (94.46%), the vast majority of correlations were positive and only 926 correlations (5.54%) were negative. 

Out of the 238 lncRNAs with a significant RIF score in the network, 22 were also categorized as a DE or QTL locus (see Supplement 4). A total of 17 lncRNAs had a network connection with at least 10 annotated genes with an official gene symbol in the bovine genome annotation (Supplement 5). In order to also account for regulatory lncRNAs with high metabolite or exceptionally high gene connectivity, the following additional lncRNAs were selected: five lncRNAs that were correlated with over ten annotated genes and over ten metabolites, and five lncRNAs that showed a connectivity with more than 50 annotated genes. One lncRNA passed both filtering steps (Supplement 5). Finally, 26 hub lncRNAs remained that were of interest regarding their associated interacting networks. These lncRNAs are candidates that probably have a regulatory potential for modulating biological pathways linked to divergent feed efficiency. One of these hub lncRNAs (*MSTRG.16058*) was connected with 14 RNA genes (including snRNAs and snoRNAs), which had escaped filtering. Due to its clear involvement in ribosomal RNA expression and unsuccessful mapping of these genes in Ingenuity Pathway Analysis (IPA), this lncRNA was excluded from further analyses. 

### 2.7. Natural Antisense Transcripts

Out of the 238 lncRNAs with a significant RIF score (key lncRNAs), 237 had a predicted positional interaction partner locus in the FEELnc results. Thereof, 119 lncRNA loci (50%) were in antisense orientation and overlapped with an annotated locus that was termed as the most likely interaction partner (isbest score = 1 according to FEELnc). These lncRNAs can be designated as natural antisense transcripts (NATs). Of these 119 antisense lncRNA–sense partner locus pairs, 44 (18.49%) had a significant correlation in the PCIT analysis (Supplement 6). The vast majority (42 out of 44) were positive correlations and two pairs were negatively correlated. Negative correlations were found for the lncRNA *MSTRG.13915* and *AZGP1* (*Zinc-alpha-2-glycoprotein*, r = −0.67) and the lncRNA *MSTRG.5787* and *EPRS* (*Glutamyl-Prolyl-TRNA Synthetase 1*, r = −0.51). A total of eight lncRNA-partner locus pairs were found to have a positive co-expression with r > 0.9. The pairs *MSTRG.5042* and *APOA1* (*Apolipoprotein A*, r = 0.98) and *MSTRG.7472* and *HP* (*Haptoglobin*, r = 0.97) displayed the strongest correlation coefficients (see Supplement 6). Regarding the ratio of expression levels of the *cis*-interaction partner gene and the corresponding antisense lncRNA, we observed pronounced differences with a minimal expression ratio of 0.21 and a maximum ratio of 392.77 (mean = 40.34, SD = 61.12). Furthermore, the ratio of expression levels is not necessarily dependent on or in a linear relationship with the general expression level of the two loci, which suggests that this observation is more than random noise (Supplement 6). Only in two cases out of the 44 antisense lncRNA-sense partner locus pairs did the antisense lncRNA have a higher expression level than that of the respective *cis*-interaction partner gene and in such cases the expression ratio was comparatively low (below 0.5). 

### 2.8. Characteristics of Key Regulatory Long Non-Coding RNAs in the Co-Expression Network

Out of the 26 hub lncRNAs (see Supplement 4), three coincided with a known QTL for RFI and 16 were differentially expressed between the efficiency groups. Two of these lncRNAs were both DE and overlapped with a QTL: *MSTRG.4802* and *MSTRG.4839*. In addition, we detected two hub lncRNAs, *MSTRG.3808* and *MSTRG.7798*, to be already annotated as lncRNAs in Ensembl release 97 (*ENSBTAG00000048400* and *ENSBTAG00000053946,* respectively). Both lncRNAs, which were included in our co-expression network, were also predicted by FEELnc to be partner loci to other lncRNAs in the dataset. 

The screening for potential *cis*-actions of the 26 hub lncRNAs, i.e., a significant PCIT correlation of |r| ≥ 0.65 with a locus no farther than 1 Mb away, showed that potentially interacting neighbouring loci could be predicted for 18 out of the 26 hub lncRNA loci. With a total of 45 interactions found, each hub lncRNA had 2.5 *cis*-interactions on average. 

Again, the highest correlation coefficients between lncRNA and *cis*-interaction partners were found for *MSTRG.5042* and *Apolipoprotein A1* (*APOA1*, r = 0.98) and for *MSTRG.7472* and *Haptoglobin* (*HP*, r = 0.97). Two of the 26 hub lncRNAs stood out because of their strong wiring with plasma metabolites: *MSTRG.4390* and *MSTRG.5042* were significantly correlated (*p* ≤ 0.01, |r| ≥ 0.65) with 44 and 45 metabolites, respectively. Both hub lncRNAs are also correlated with each other (r = 0.80) and shared 24 loci correlation partners and 42 metabolite correlations. Out of these 42 shared metabolite correlation partners, five were differentially abundant between both groups (q (Benjamini-Hochberg) ≤ 0.05 and |log_2_FC| ≥ 1): 10-heptadecenoate (17:1n7), 4-hydroxyglutamate, 9-hydroxystearate, succinate and tetradecanedioate (see Supplement 3).

**Figure 5:**
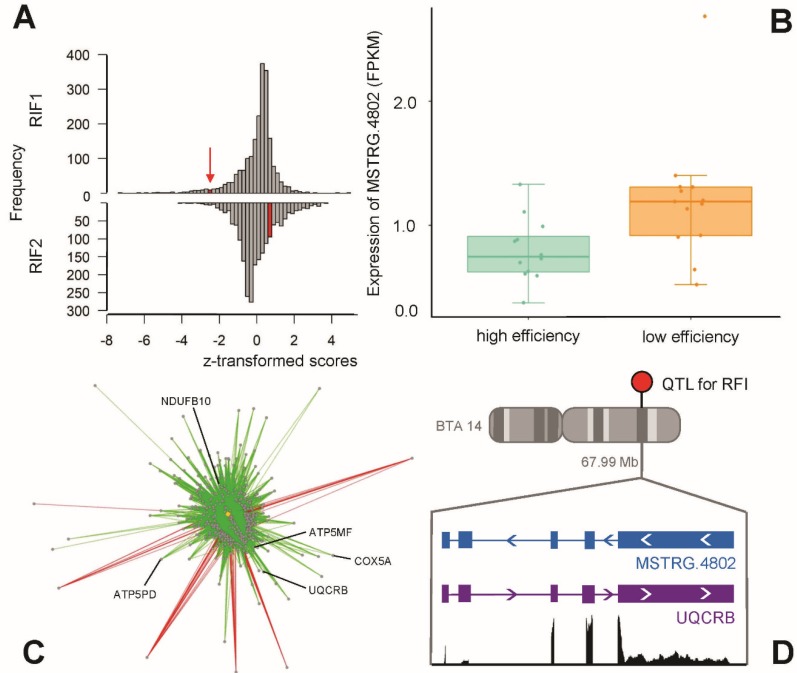
Key lncRNA *MSTRG.4802* with (**A**) a significant RIF1 score, (**B**) differential expression between bulls of high and low feed efficiency, (**C**) high connectivity in a co-expression network, and (**D**) antisense direction to protein-coding gene *UQCRB* on bovine chromosome BTA14 at 67.99 Mb, coinciding with a remapped quantitative trait locus (QTL) for residual feed intake (RFI).

Because of its multi-categorization (hub lncRNA, DE and QTL locus, *cis*-interaction), we selected *MSTRG.4802* (see Figure 5) for a more detailed analysis of its regulatory function with regard to associated biological pathways. Due to their strong intertwining and apparent connection to plasma metabolite levels, *MSTRG.4390* and *MSTRG.5042* have also received more focus. *MSTRG.5042*, along with *MSTRG.7472*, are relevant due to their strong *cis*-interaction with the corresponding antisense oriented protein-coding gene. Table 2 summarizes positional and structural information for these top four potentially regulatory lncRNAs and condenses results from the differential expression analysis, FEELnc application, and the screening for *cis*-interactions. These lncRNAs have been classified by FEELnc as antisense lncRNAs transcribed in the opposite orientation to their partner genes and can be regarded as NATs. The expression levels of the four hub lncRNAs and their antisense protein-coding partner genes are depicted in Figures S1–S4.

### 2.9. Pathway Enrichment Analysis

In order to detect generally enriched pathways in the liver transcriptome between the two efficiency groups, the DE genes were submitted to Ingenuity Pathway Analysis (IPA). The pathway PPARα/RXRα activation was significantly enriched (*p* ≤ 0.01, equaling −log_10_(*p*) ≥ 2.0) (downregulated in highly efficient bulls (−log_10_(*p*) = 6.12, z-score = −0.707), as was the pathway of VDR/RXR activation (−log_10_(*p*) = 3.06, z-score = −1.0). A slight upregulation of the NRF2-mediated oxidative stress response for high efficiency bulls was also detected (−log_10_(*p*) = 2.83, z-score = 0.447).

Focusing on transcriptional upstream regulators, we observed the strongest inhibition in the high efficiency group for the peroxisome proliferator-activated receptor gamma coactivator 1-alpha (PPARGC1A, activatio*n* = −2.268, *p* = 1.3 × 10^−3^) and the strongest activation for the hypoxia-inducible factor 1-alpha (HIF1A, activatio*n* = 2.348, *p* = 3.21 × 10^−3^). Detailed results for enriched canonical pathways and upstream regulators are given in Supplement 7 and Supplement 8, respectively. 

The Ingenuity pathway enrichment analysis with genes associated with the four selected top hub lncRNAs (the NATs *MSTRG.4390*, *MSTRG.4802*, *MSTRG.5042*, *MSTRG.7472*) revealed significant enrichments of specific biological pathways. A summary of the top five enriched canonical pathways is provided in Table 3. Transcriptional upstream regulators are listed in Table 4, prioritized for results with an activation score if available.

*MSTRG*.*4802* had by far the strongest z-score (−2.236) for the pathway of oxidative phosphorylation (−log_10_(p) = 7.00), followed by mitochondrial dysfunction (−log_10_(p) = 6.02). The enrichment of both pathways was based on the correlated genes *ATP5MF, ATP5PD, COX5A, NDUFB10* and *UQCRB* encoding protein members of mitochondrial respiratory chain complexes. The gene *ubiquinol-cytochrome c reductase binding protein* (*UQCRB*) was predicted by FEELnc to be the positional interaction partner for *MSTRG.4802*, which was confirmed by the finding of a *cis*-interaction. The lncRNA *MSTRG.4802* was found in the antisense direction to its interaction partner and both loci displayed a positive correlation of their expression levels with r = 0.69. The abovementioned upstream regulators PPARGC1A (*p* = 6.2 × 10^−3^) and HIF1A (*p* = 1.44 × 10^−3^) were detected to be significant for genes correlated with *MSTRG.4802* (see Supplement 7), as well as the paralogue transcription regulator PPARGC1B (*p* = 3.0 × 10^−3^). 

Of all performed enrichment analyses, *MSTRG.7472* had the overall lowest *p*-value (−log_10_(p) = 11.2) for the pathway of acute phase response signaling, which was downregulated in the high efficiency group (z-score = −0.378). One of the major genes involved in this pathway is *haptoglobin* (*HP*), which we predicted to be in *cis*-interaction with lncRNA *MSTRG.7472*. In addition, the pathway unfolded protein response was found to be upregulated in highly efficient bulls (−log_10_(p) = 6.82, z-score = 0.447) for *MSTRG.7472*. One of the correlated genes, *STAT3*, was also found to be a downregulated transcription regulator (activatio*n* = −0.877, *p* = 6.51 × 10^−5^). Again, an upregulation of HIF1A (activatio*n* = 1.932, *p* = 3.21 × 10^−3^) could be registered, as well a positive activation of hepatocyte nuclear factor 1 homeobox a (HNF1A, activatio*n* = 1.114, *p* = 1.77 × 10^−6^) (Supplement 8). 

The lncRNAs *MSTRG.4390* and *MSTRG.5042* were highly correlated with each other (r = 0.80). The analysis showed that, based on their correlation partners, they were enriched for functionally related pathways: fatty acid ß-oxidation (−log_10_(*p*) = 5.56, z-score = 1) was upregulated for *MSTRG.4390* in highly efficient animals and *MSTRG.5042* showed an enrichment for the TCA cycle II (−log_10_(*p*) = 3.48, no z-score) in this experimental group. The analysis of potential upstream regulators revealed the same strongest transcriptional regulators for both lncRNAs: a downregulation of promyelocytic leukemia (PML; *MSTRG.4390*: activatio*n* = -2.433, *p* = 1.22 × 10^−6^, *MSTRG.5042*: activatio*n* = −2.000, *p* = 1.89 × 10^−3^) and an upregulation of PPARGC1B (*MSTRG.4390*: activatio*n* = 2.177, *p* = 4.51 × 10^−7^, *MSTRG.5042*: activatio*n* = 2.000, *p* = 8.73 × 10^−5^) in the high efficiency group could be observed (Table 4). 

The analysis of both lncRNAs and their correlation partners combined showed a significant enrichment for valine degradation (-log_10_(*p*) = 5.18, z-score = 0), followed by the pathways that had been detected on an individual basis as well: fatty acid ß-oxidation (−log_10_(*p*) = 4.74, z-score = 1.00) and the TCA cycle II (-log_10_(*p*) = 3.37, no z-score). Analogously, to the individual analysis of potential upstream regulators, the strongest activation for transcription regulators was observed for PPARGC1B (activatio*n* = 2.177, *p* = 6.26 × 10^−6^) and PML was significantly inhibited (activatio*n* = -2.433, *p* = 2.68 × 10^−5^) in animals of high efficiency (see Supplement 7 and Supplement 8).

## 3. Discussion

We studied crossbred F_2_-bulls (Charolais x Holstein Friesian) with divergent feed efficiency and fat deposition at a transcriptomic (liver) and metabolomics (blood plasma) level and integrated these data to identify lncRNAs and predict their potential biological function through biological pathway enrichment analyses. Using the bioinformatics lncRNA prediction tool FEELnc [22], which has been applied to determine lncRNAs in different species, including dogs [44], chicken [45], cattle [30,36] and pigs [46], we have identified 3590 lncRNA loci expressed in the liver transcriptome. 

In a previous study, our group employed the herein presented pipeline, which applied a systems biology approach combining RIF and PCIT algorithms with biological network prediction to identify potential key regulatory lncRNAs in a tissue- and sex-overarching approach [36]. However, other studies have shown that many lncRNAs are tissue-specific in their expression pattern [47]. To better understand the function of lncRNAs and their interactions in the liver, we have now focused on this single organ due to its relevance in the context of metabolism [38] and the immune system [48]. 

We therefore adjusted the pipeline, especially regarding the loci set prioritization: the category of tissue-specific loci was excluded and instead potential positional partner loci of lncRNAs, as predicted by FEELnc, were included. Furthermore, we lowered the minimal expression threshold to at least 0.1 FPKM in at least six animals of one group. In contrast to the previous study, we used raw FPKM values for calculations instead of log-transformed values. This step presented itself as necessary to account for the relatively low abundance of lncRNAs compared with mRNAs in the transcriptome [37,49]. Indeed, in our study, 2335 out of the 3590 lncRNA loci (65%) had an average expression level in the liver of less than 1 FPKM. 

The prediction of potential biological functions of the identified key lncRNAs was based on the premise that they were involved in the same biological pathway as their correlated partner loci or metabolites. This guilt-by-association heuristic, in which correlating genes or metabolites are used to perform enrichment analyses for biological pathways and then to infer functional involvement for novel, non-coding elements has already been applied to miRNAs and lncRNAs, e.g., [27,32], and [30]. When interpreting the results of such analyses, it should be kept in mind that these predictions heavily depend on the statistical method used to calculate the correlation coefficients [50]. The PCIT algorithm that we applied in our study ensures that the detected pairwise loci correlations are independent of any other third locus in the dataset [35]. 

Up to now, the combined application of the RIF and PCIT allowed for the discovery of regulatory genomic elements in cattle with regard to a variety of phenotypes: e.g., feed efficiency [51], puberty [52,53], as well as the mineral content [54], intramuscular fat content [55] and fatty acid composition in muscle [56]. Our study showed that the functional prediction of lncRNAs with potential regulatory activity in cattle that differed in their phenotypes in terms of feed efficiency, pointed towards their involvement in immunological pathways, the TCA-cycle, fatty acid β-oxidation, and mitochondrial function.

### 3.1. LncRNAs Participating in Fatty Acid β-Oxidation and TCA-Cycle

The relevance of mitochondrial function and energy metabolism for feed efficiency was underlined by the key lncRNAs *MSTRG.4390* and *MSTRG.5042* and their respective pathway enrichments for fatty acid β-oxidation and the TCA-cycle. In the mitochondria, the fatty acids are broken down to produce acetyl-CoA that then enters the TCA cycle. The β-oxidation is MTP-dependent (mitochondrial trifunctional protein), which is encoded by the genes *HADHA* and *HADHB*. The latter was part of our prioritized loci set because it was predicted as the positional interaction gene of lncRNA *MSTRG.2669*, but it turned out to be significantly correlated (r = 0.7153) with *MSTRG.4390*. Though no differential abundance was found for carnitine or acetylcarnitine, which are indicative of a challenged β-oxidation when decreased [57], a number of long-chain fatty acids (e.g., stearoyl carnitine, palmitoyl carnitine, docosapentaenoate) was positively correlated with *MSTRG.4390* expression, along with the related enzyme encoding gene *ACSL1*. Both *MSTRG.4390* and *MSTRG.5042* shared most of their correlation partners, including fatty acids, which suggests a common biological function. However, only *MSTRG.5042* correlated with all three successive TCA cycle products: succinate, fumarate, and malate. Analogous to these findings, Wang and Kadarmideen [58] also found an enrichment for the citrate cycle in an integrative study of metabolomics and transcriptomic data in cattle divided into high and low residual feed intake. 

A definitive functional prediction for *MSTRG.5042* remained challenging, because its strongest associations (r > 0.9) were with its *cis*-partners *APOA1* (*Apolipoprotein A1*) and *MAT2A* (*Methionine Adenosyltransferase 2A*). The protein encoded by *MAT2A* catalyses the production of S-adenosylmethionine from methionine. While *MAT2A* had higher expression levels in animals of high feed efficiency, methionine itself was of significantly higher abundance in plasma in bulls of low feed efficiency (high RFI). *APOA1* was downregulated in bulls of low efficiency, which is in accordance with findings of Gondret et al. [59] in pigs and Zhuo et al. [60] in chickens. It is noteworthy that the lncRNA *MSTRG.5042* was exactly in the antisense position to *APOA1*, but displayed a 50-fold lower average expression. We found that PPARGC1B, a key regulator of mitochondrial biogenesis [61], is the most strongly activated upstream regulator (z-score = 2.177, *p* = 6.26 × 10^−6^) when comparing animals of high efficiency with low efficiency animals, which is supported by the findings of Vigors et al. [62] in pigs. 

### 3.2. LncRNA Linked to Mitochondrial Function and Energy Metabolism

Exploring the potential regulatory impact of the hub lncRNAs revealed that they might modulate mitochondrial processes and energy metabolism. In our study, the enrichment hits for lncRNA *MSTRG.4802* suggest its involvement in oxidative phosphorylation and mitochondrial dysfunction. *MSTRG.4802* was particularly interesting, because it did not only have a significant RIF1 score—and thereby a predicted high regulatory potential—but was also DE with a significantly lower expression in high efficiency bulls. In addition, its *cis*-interaction partner *UQCRB* (*Ubiquinol-Cytochrome C Reductase Binding Protein*) also displayed a lower expression level in animals of high feed efficiency. *UQCRB*, which is fundamental for the functioning of the mitochondrial respiratory chain complex III [63], is on the opposite strand and in complete overlap with *MSTRG.4802*. Interestingly, this locus falls within a remapped QTL region for RFI as well [4], which supports its putative relevance in the regulation of the related biological processes. 

### 3.3. LncRNA Associated with Immunological Functions

There is a tight relationship between the animal’s immune response and its performance in feed efficiency or growth-related traits. Although not DE in our dataset, the correlation of *MSTRG.7472* with *HP*, *LBP, SOCS3* and *SAA2* indicates that this lncRNA is functionally involved in the acute phase signaling. Already in early life stages, inflammation negatively affects growth rates and the average daily gain in feedlot calves [64]. Subsequently, at puberty, gene modules that were associated with feed efficiency in bulls showed enrichments for an immune and an inflammatory response, whereby the authors had reasons to assume that this was due to a bacterial infection of the liver [39]. Mukiibi et al. [41] assessed the liver transcriptome of bulls—similar in age to our cohort—in different breeds and found the acute phase signaling pathway to be among the top enrichment hits in Angus steers of divergent growth performance. 

### 3.4. LncRNAs Putatively Involved in Gluconeogenesis 

As Ingenuity Pathway Analysis is deeply rooted in human research, biological processes and pathways that are specific to other species might therefore be overlooked. We considered it noteworthy that *MSTRG.4390* and *MSTRG.5042* both correlated with the gene *PCK1* at expression level and that *MSTRG.5042* expression also correlated with that of *FBP1*. Both *PCK1* and *FBP1* occupy key roles in gluconeogenesis, a biological pathway that is particularly important for the energy balance in cattle [65]. The correlation of *MSTRG.504*2 with the metabolite glycerol supports the assumption that these lncRNAs might be involved in the regulation of hepatic gluconeogenesis in cattle [66]. Additionally, we found lactate to be differentially abundant and at significantly higher levels in the plasma of highly efficient animals. The available amount of the glucogenic precursors lactate and glycerol, next to glucogenic amino acids and volatile fatty acids, substantially influences the hepatic glucose production [66]. In this context, we found that the high-connectivity key lncRNA *MSTRG.9118* was co-expressed with *G6PC,* encoding the enzyme that controls the glucose release in hepatocytes and thereby plays a central role in this biological pathway [67]. *MSTRG.9118* is also antisense oriented to *G6PC.*

### 3.5. LncRNAs as Natural Antisense Transcripts

The above-mentioned four hub lncRNAs (*MSTRG.4390, MSTRG.4802, MSTRG.5042, MSTRG.7472*) lie in antisense orientation to and almost completely overlap with a protein-coding gene on the opposite strand. Furthermore, all four hub lncRNAs were positively co-expressed with their *cis*-partner locus. The observation of nearly complete or perfect antisense overlaps between the paired protein-coding genes and non-coding RNAs has already been described and reviewed for natural antisense transcripts (NATs) by Latgé et al. [68]. Our observation of predominantly positive correlations between key lncRNAs and the paired locus on the opposite strand confirmed the findings of Wenric et al. [17]. The authors found that strong negative correlations (r < −0.4) between the mirroring pairs were rare and the correlation coefficients ranged between 0.431 and 0.533 [17]. Indeed, we only found two strong negative correlations between key antisense lncRNAs and the overlapping paired partner locus. We could also confirm strong differences in expression levels between the non-coding NATs and their protein-coding partners, although not as strong as described by Wenric et al. (up to a 1000-fold). Indeed, the observed expression ratios of partner gene expression level divided by antisense lncRNA expression level were rather variable and ranged from 0.21 to 392.77. Only in two exceptional cases of *cis*-interactions of NAT lncRNAs (out of 44) did these have higher expression levels than their *cis*-partner gene, and in both cases, the expression ratio was below 0.5. As reported by Napoli et al. [69], NATs have been found to be implicated in multiple regulatory mechanisms, including RNA masking, alternative splicing and chromatin remodelling. A conceivable function of our key lncRNAs, which are positively correlated with their associated antisense locus partner at expression level, could be the stabilization of the corresponding paired transcript. The stabilization might occur by protecting the transcript from degradation, binding to miRNAs or corrosive post-transcriptional processes [70]. Such lncRNAs with potential protective properties would easily have been overlooked in the past before the introduction of stranded RNA sequencing libraries in 2008 [71].

## 4. Materials and Methods 

### 4.1. Animals

The bulls in our study were part of a F_2_-population of a Charolais x Holstein Friesian cross (SEGFAM [72]). The animals were bred and raised at the Leibniz Institute for Farm Animal Biology (FBN) in Dummerstorf (Germany) and kept under standardized housing conditions, as previously described by Eberlein et al. [73] and Widmann et al. [74]. The bulls’ individual feed intake was measured daily, and body weight was recorded on a monthly basis. Animals were slaughtered at 18 months of age and the carcasses underwent detailed dissection, including measurements for intramuscular (IMF) and carcass (CF) fat percentage. The bulls were split into groups of high or low efficiency depending on their residual feed intake (RFI) in the last month of life, their IMF in *M. longissimus dorsi* and their CF percentage. Bulls were assigned to the high efficiency group if they had a low RFI (at least one standard deviation (SD) below average) and a lower CF as well as a lower IMF than the population mean (CF: mea*n* = 16.5% ± 4.0%; IMF: mea*n* = 3.67% ± 1.76%; *n* = 246). All animals had to have a positive daily weight gain and no less than the population average minus one SD. Accordingly, bulls were grouped to low efficiency if they had a high RFI (at least one SD above average), and a higher CF and IMF than the mean (see Table 5). Archer’s formula [75] was used to calculate the individual RFI, which equals the bulls’ energy intake while accounting for the average daily weight gain and metabolic mid-weight (average body weight during the last month of life raised to the power of 0.75). For the current study, out of 246 deeply phenotyped F_2_-bulls, 26 bulls were selected with extremely high (*n* = 13) or low efficiency (*n* = 13).

All experimental procedures were carried out according to the German animal care guidelines and were approved (27 March 2003) and supervised by the relevant authorities of the State Mecklenburg-Vorpommern, Germany (State Office for Agriculture, Food Safety and Fishery; LALLF M-V/TSD/7221.3-2.1-010/03).

### 4.2. Plasma Metabolites

Blood samples were taken on the day of slaughter before transit to the slaughterhouse and holistic metabolite profiles with 640 biochemical compounds and molecules in plasma were established by Metabolon Inc. (Durham, NC, USA, https://www.metabolon.com/). With ultra-high-performance liquid chromatography and tandem accurate mass spectrometry (UHPLC/MS/MS) methods, compounds and derivatives of eight different metabolite classes were determined: amino acids (*n* = 167), carbohydrates (*n* = 27), cofactors and vitamins (*n* = 19), energy (*n* = 10), lipids (*n* = 278), nucleotides (*n* = 36), peptides (*n* = 35), and xenobiotics (*n* = 68). As animal B002 (high efficiency group) clustered unexpectedly within the inefficient group in the transcriptomic analysis, this animal was excluded from further metabolomics analysis steps. 

For differential abundance analysis of metabolites in the blood plasma, the R-package MetaboDiff [76] was used and the author’s instructions were closely followed. As recommended, metabolites with more than 40% missing cases were excluded and for the remaining metabolites, missing values were imputed with the k-nearest neighbor algorithm. A total of 552 metabolites remained in the dataset, which was then normalized using a variance stabilization transformation. For the comparison of the high and low efficiency group, a Student’s *t*-Test was applied, and *p*-values were corrected for multiple testing with the Benjamini–Hochberg procedure [77]. 

### 4.3. Sampling, RNA Isolation, Library Preparation, and Sequencing

Immediately after slaughter and dissection, tissue samples were taken from the liver (*Lobus caudatus*), shock frozen in liquid nitrogen and then stored at −80 °C. For RNA extraction, the samples were ground in liquid nitrogen and 30 mg were subjected to an on-column-purification with the NucleoSpin RNA II kit (Macherey and Nagel, Düren, Germany), which included a DNase digestion to remove genomic DNA. RNA was subsequently tested for remaining DNA residues and further cleansed, if necessary, according to Weikard et al. [78]. The RNA concentration and integrity were measured with a Qubit Fluorometer (Invitrogen, Karlsruhe, Germany) and a 2100 Bioanalyzer Instrument (Agilent Technologies, Waldbronn, Germany). From 1 µg of total RNA per sample, stranded, ribodepleted and indexed libraries were prepared with the TruSeq Stranded RNA-Ribo-Zero H/M/R Gold Kit (Illumina, San Diego, CA, USA). Paired-end reads were sequenced (2 × 100 bp) in a multiplexed design on a HiSeq 2500 Sequencing System (Illumina). 

### 4.4. Alignment and Assembly

Raw reads were subjected to quality control with FastQC [79], adapter trimming with Cutadapt v.1.6.1 [80] and thereafter quality trimming with Quality Trim v. 1.6.0 [81]. For quality trimming, the sequence start was also processed (option -s), the maximum number of missing bases (N) was set to 3, and the minimum base quality was set to 15. In a guided alignment, the reads were then mapped with HISAT2 v.2.1.0 [82] to the latest bovine reference genome ARS-UCD1.2 [83] with Ensembl annotation release 97 [84]. The sorting and indexing of BAM files were performed with samtools v.1.6 [85] and Stringtie v.1.3.4d [86] was used for the individual assembly while using the reference genome and annotation in a guided approach. For this study, we created a project-specific annotation with Stringtie merge (default settings for Stringtie merge and a minimal read alignment per exonic base (-c) of 15). To this end, we made use of the bovine reference genome, the 26 bull liver samples, as well as 178 other samples available from a previous study [36]. These samples included 26 liver samples from cows of the same resource population, as well as muscle (*n* = 52), jejunum (*n* = 48) and rumen (*n* = 52) samples of these cows and the bulls used in the present study. 

The merged annotation was checked for plausibility, i.e., the number of exons for each transcript and the number of transcripts for each locus. We excluded loci that had over 20 transcripts, unless one of these transcripts was already annotated, in which case only that particular transcript was kept for the locus. In the reference annotation (Ensemble release 97), the maximum number of exons per transcript was 173 and therefore we set a cut-off threshold of 200 exons per transcript. Transcripts with more than 200 exons were excluded from the merged annotation, except for two transcripts overlapping with the gene *titin,* which is highly expressed in muscle tissue and has been annotated with 335 exons in NCBI (National Center for Biotechnology Information, annotation release 106). 

The transcriptome dataset examined in this study was already used in a previous study ([36], aligned to UMD.3.1, Ensembl annotation release 92) and is stored in the Functional Annotation of Animal Genomes (FAANG) database (https://data.faang.org/dataset) under project number PRJEB34570.

### 4.5. Long Non-Coding RNA Prediction and Fragment Counting

The computational identification of lncRNAs was carried out with FEELnc [22], while making use of the merged annotation and the bovine reference genome and annotation ARS-UCD1.2. (Ensembl 97). Annotated loci of the protein coding biotype were excluded, and the minimal transcript length was kept at the default of 200 nt. To reduce the number of false positives, monoexonic transcripts were discarded, unless they were in antisense localization. The coding potential for all remaining transcripts was determined in shuffling mode. 

Except for the differential expression analysis, fragments per kilobase per million mapped reads (FPKM) were used in all further analysis steps. These were calculated based on fragment counts derived with featureCounts [87]. All loci needed to have a minimal expression of at least 0.1 FPKM in at least six animals of one experimental group. The expression threshold was deliberately set this low in order to keep as many predicted lncRNAs in the dataset as possible. Loci that were annotated as ribosomal, spliceosomal, metazoan or Y-RNA genes were generally discarded.

### 4.6. Loci Set Prioritization

To enable the construction of meaningful co-expression networks, we compiled a list of prioritized loci, which included loci that belonged to at least one of the following four categories: predicted lncRNA (lncRNA), potential interaction partner of the lncRNA (partner locus), overlapping or in close proximity of up to 3 Mb of a QTL (QTL locus), and differentially expressed between the groups of high and low efficiency (DE locus). 

Loci were included in the ‘lncRNA’ category if one of the locus’ transcripts was predicted as lncRNA using FEELnc and the minimal expression threshold was exceeded. Loci were included in the category ‘partner locus’ of the prioritized loci set if FEELnc predicted them to be positional interaction partners and rated them ‘best choice’ with a score of 1. FEELnc determines the most likely positional interaction partner for a lncRNA based on its physical genomic position relative to the nearest locus. The best choice thereby is a locus that overlaps with the lncRNA, preferentially at an exon, and if no overlapping locus can be found, the closest neighbor is chosen instead. 

Loci were included in the category ‘QTL locus’ if they were minimally expressed and overlapped with or were no farther away than 3 Mb from a QTL for residual feed intake (RFI) in cattle. QTLs were downloaded from the Animalgenome QTL database (https://www.animalgenome.org/cgi-bin/QTLdb/BT/index, accessed 10 October 2019) and only QTL based on SNP array studies were kept. The QTL positions were then remapped to the new reference genome ARS-UCD1.2 with the NCBI Genome Remapping Service and default options (https://www.ncbi.nlm.nih.gov/genome/tools/remap, accessed on 22 November 2019). 

The differential expression analysis was performed with the R-package DESeq2 [88]. Cluster analysis revealed unexpected clustering of animal B002 in a PCA-plot based on read counts. Due to pathological findings in the liver, this animal was excluded from all further analyses. The model for differential expression analysis included the efficiency group; an effect of year of slaughter or birth could not be included because all animals of the high efficiency group were born between 2002 and 2007 and all animals of the low efficiency group were born between 2008 and 2011. Loci were considered significantly differentially expressed (DE) if they were minimally expressed and withstood a correction for multiple testing with the Benjamini–Hochberg [77] procedure (adjusted *p*-value (*q*) ≤ 0.1). 

### 4.7. Regulatory Impact Factor Analysis

The regulatory impact factor (RIF) algorithm of Reverter et al. [34] is designed to detect loci with high regulatory potential in a prioritized loci set while contrasting two biological conditions or groups. The analysis makes use of two metrics: RIF1 and RIF2. A high RIF1 score was attributed to lncRNAs that were co-expressed with abundant target loci (DE, QTL, partner) in both efficiency groups. A high RIF2 score was assigned to lncRNAs if they were strongly correlated with a target locus in one group but displayed no or a reversed correlation to the same target locus in the other group. Since some lncRNAs were also categorized as DE, QTL or partner loci, they could also be targets in the RIF analysis. RIF scores were standardized with a z-transformation and lncRNAs with either a RIF1 or RIF2 score of ≥ 1.96 were deemed significant, which corresponds to a significance threshold of *p* ≤ 0.05 in a t-test. Subsequently, lncRNAs with a significant RIF score (key lncRNAs) were closely scrutinized in the co-expression networks. 

### 4.8. Partial Correlation and Information Theory

The partial correlation and information theory (PCIT [35]) calculates pairwise correlations between loci while accounting for the influence of a third locus. Unlike likelihood-based approaches, which invoke a parametric distribution (e.g., normal) assumed to hold under the null hypothesis and then a nominal *p*-value (e.g., 5%) used to ascertain significance, PCIT is an information theoretic approach. Its threshold is an informative metric, in this case the partial correlation after exploring all trios in judging the significance of a given correlation, which might then become a connection when inferring a network. It thereby tests all possible three-way combinations in a dataset and only keeps correlations between loci if they are significant and independent of the expression of another locus, whereas no hard threshold is set for the correlation strength. The significance threshold for each combination of loci depends on the average ratio of partial and direct correlation [35]. The set of prioritized loci that was subjected to the RIF analysis was also used for the PCIT. 

### 4.9. Correlation of Plasma Metabolites with Key LncRNAs

A Pearson correlation coefficient was calculated with the function rcorr of the Hmisc R-package [89] for all key lncRNAs (significant RIF score) and plasma metabolites. The data curation was independent from the differential abundance analysis of metabolites and a lower number of missing cases was accepted for the correlation analysis. The raw metabolite values were filtered for metabolites with less than five missing cases and missing values imputed with the minimum observation, assuming that the missing value was due to a value below the detection limit and not a technical error. The values were then scaled with the scale-function in R (without centering). Correlations were considered significant if they had a *p* ≤ 0.01. 

### 4.10. Natural Antisense Transcripts

The results from FEELnc were filtered for key lncRNAs (significant RIF score) that overlapped with a predicted positional interaction partner locus on the opposite strand (antisense direction and a distance of 0 bp to the partner locus). LncRNAs that are in antisense position to another gene have been described as natural antisense transcripts (NATs) in the literature before [17] and fall into the category of *cis*-interaction partners. We wanted to screen for valid *cis*-interactions, meaning a correlation in expression and not a mere positional neighborhood. To this end, we checked for significant PCIT correlations between the antisense lncRNAs and the respective partner loci, regardless of correlation strength or direction (positive or negative).

### 4.11. Selection of Hub Key lncRNAs in Co-Expression Network

The visualization of the co-expression network was realized in Cytoscape 3.6.1 [90]. All significant PCIT correlations with a minimum strength of |r|≥ 0.65 between lncRNAs with a significant RIF score and any other locus from the prioritized loci set were included. Additionally, significant correlations between the above-mentioned lncRNAs and plasma metabolites were also included if they had a minimal correlation strength of |r|≥ 0.65. We filtered for lncRNAs with a significant RIF score that were correlated with at least 10 annotated genes, having an official gene symbol available and not predicted to be a lncRNA. To further narrow down the selection to impactful lncRNAs, we filtered for lncRNAs that fulfilled either of the following three criteria: I) categorization as a DE or QTL locus, II) additional correlation with at least 10 metabolites, or III) exceptionally high connectivity with >50 annotated genes with an official gene symbol in the bovine genome annotation. LncRNAs that fulfilled these criteria were labelled key lncRNAs.

### 4.12. Cis-Action of Hub LncRNAs

In addition to the screening for NATs, we searched for *cis*-interaction partners for hub lncRNAs in a larger radius. All loci within a physical distance of up to 1 Mb and with a correlation significant according to PCIT and substantial in magnitude such that |r| ≥ 0.65 were considered for each individual hub lncRNA. Since the lncRNA prediction in FEELnc works in a transcript-based manner, only the transcript of a locus that was actually predicted to be non-coding was considered. 

### 4.13. Pathway Enrichment Analysis

In order to discern the probable biological functions of hub lncRNAs, we conducted pathway enrichment analyses with significantly and substantially correlated loci and metabolites (|r| ≥ 0.65) for each of them. Additionally, to investigate which biological pathways are generally to be addressed for our animal material and phenotype, an enrichment analysis was done for all DE between the high and low efficiency group. The list of metabolites and genes and their logged fold changes were submitted to the Ingenuity Pathway Analysis (IPA: QIAGEN, Inc., http://www.qiagenbioinformatics.com/products/ingenuity-pathway-analysis) [91]. Pathways were considered significantly enriched at a *p*-value of *p* ≤ 0.05 equalling a −log_10_(*p*-value) of 1.3. The same significance threshold was applied to upstream regulators in the pathway enrichment analyses. 

## 5. Conclusions

With this study, we enlarged the catalogue of lncRNAs from bovine liver, identified hub lncRNAs that are potentially involved in biological processes and pathways modulating feed efficiency in bulls and made first predictions contributing to their functional annotation. Our results underline the importance of immunological pathways and metabolic pathways associated with mitochondrial processes of the metabolic phenotype related to feed efficiency in bulls and suggest a possible regulatory function of key lncRNAs with regard to their modulating and fine-tuning role within these biological pathways.

A substantial proportion of the identified lncRNAs fall into the category of natural antisense transcripts, which most likely perform a stabilizing function with respect to mRNAs transcribed from the opposite strand. This function needs to be validated by further studies. To what extent these lncRNAs and the associated biological processes and pathways are also relevant in cows or bulls at other life stages requires further investigations. 

## Figures and Tables

**Table 1:** RNA sequencing, alignment, and mapping statistics.

	Sequencing Depth [Read Pairs]	Alignment to ARS-UCD.1.2 (%)	Mapping to Project-Specific Annotation (%)
Mean	49,831,770	98.72	85.98
SD	5,588,004	0.26	1.40

SD = standard deviation.

**Table 2:** Characteristics of four hub lncRNAs with relation to feed efficiency in bulls.

**lncRNA**	**Position**				**Structure**		**Expression (FPKM ^3^)**			**Differential Expression Analysis**		
**Locus ID**	**BTA ^1^**	**Start bp ^2^**	**End bp**	**Strand**	**Number Exons**	**Exonic Length**	**Mean**	**Mean High Efficiency Group**	**Mean Low Efficiency Group**	**Log_2_FC ^4^**	***p*-Value**	**Adjusted *p*-Value (BH ^5^)**
*MSTRG.4390*	14	518,688	534,106	-	2	20,919	2.586	2.672	2.507	0.0661	0.501	0.796
*MSTRG.4802*	14	67,986,656	67,991,285	-	5	806	1.009	0.798	1.205	-0.6310	0.004	0.091
*MSTRG.5042*	15	27,503,347	27,512,980	+	7	3,002	0.843	1.044	0.658	0.6330	0.043	0.287
*MSTRG.7472*	18	39,037,005	39,043,726	+	7	1,920	11.200	11.016	11.370	-0.1053	0.886	0.966
**lncRNA**	**FEELnc Analysis**							***cis* Action**				
**Locus ID**	**Best Potential Partner Gene**			**Direction**	**Type**	**Distance**	**Subtype Location**	**Interaction Partner Gene**			**PCIT (r) ^7^**	**Direction**
*MSTRG.4390*	*ENSBTAG00000046026*			AS ^6^	genic	overlapping	exonic	no *cis* interaction with a minimal correlation of r = 0.65				
*MSTRG.4802*	*ENSBTAG00000001521 (UQCRB)*			AS	genic	nested	exonic	*ENSBTAG00000001521 (UQCRB) MSTRG.4780 ENSBTAG00000032432 MSTRG.4798*			0.690.670.670.67	antisense sensesense sense
*MSTRG.5042*	*ENSBTAG00000002258 (APOA1)*			AS	genic	containing	exonic	*ENSBTAG00000002258 (APOA1)*			0.98	antisense
*MSTRG.7472*	*ENSBTAG00000006354 (HP)*			AS	genic	containing	exonic	*ENSBTAG00000006354 (HP)*			0.97	antisense

^1^ BTA = bovine chromosome, ^2^ bp = base pair, ^3^ FPKM = fragment per kilobase per million, ^4^ FC = foldchange, ^5^ BH = Benjamini–Hochberg, ^6^ AS = anti-sense, ^7^ PCIT (r) = correlation coefficient r from partial correlation and information theory analysis.

**Table 3:** Top 5 enriched canonical pathways for key lncRNAs related to feed efficiency.

Lnc RNA	Ingenuity Canonical Pathways	−log_10_(p)	*p*-Value	Ratio	z-Score	Molecules
***MSTRG.4390***	Fatty Acid β-oxidation I	5.56	2.75 × 10^−6^	8.89 × 10^−2^	1.00	*ACADM, ACSL1, ECHS1, HADHB*
	Palmitate Biosynthesis I (Animals)	3.52	3.02 × 10^−4^	1.67 × 10^−1^	NaN	lauric acid, palmitic acid
	Stearate Biosynthesis I (Animals)	3.52	3.02 × 10^−4^	5.00 × 10^−2^	NaN	*ACSL1*, palmitic acid, stearic acid
	Ketolysis	3.11	7.76 × 10^−4^	1.05 × 10^−1^	NaN	*HADHB*, succinic acid
	γ-linolenate Biosynthesis II (Animals)	2.91	1.23 × 10^−3^	8.33 × 10^−2^	NaN	*ACSL1*, linoleic acid
***MSTRG.4802***	Oxidative Phosphorylation	7.00	1.00 × 10^−7^	4.2 × 10^−2^	−2.236	*ATP5MF, ATP5PD, COX5A, NDUFB10, UQCRB*
	Mitochondrial Dysfunction	6.02	9.55 × 10^−7^	2.66 × 10^−2^	NaN	*ATP5MF, ATP5PD, COX5A, NDUFB10, UQCRB*
	Spermine Biosynthesis	2.16	6.92 × 10^−3^	1.43 × 10^-1^	NaN	*SMS*
	Sirtuin Signaling Pathway	1.40	3.98 × 10^−2^	6.17 × 10^−3^	NaN	*ATG3, NDUFB10*
	TNFR1 Signaling	1.32	4.79 × 10^−2^	2.00 × 10^−2^	NaN	*MADD*
***MSTRG.5042***	TCA Cycle II (Eukaryotic)	3.48	3.31 × 10^−4^	7.14 × 10^−2^	NaN	fumaric acid, L-malic acid, succinic acid
	Palmitate Biosynthesis I (Animals)	3.19	6.46 × 10^−4^	1.67 × 10^−1^	NaN	lauric acid, palmitic acid
	Glycerol Degradation I	3.12	7.59 × 10^−4^	1.54 × 10^−1^	NaN	*GK*, glycerol
	Stearate Biosynthesis I (Animals)	3.03	9.33 × 10^−4^	5.00 × 10^−2^	NaN	*ACSL1*, palmitic acid, stearic acid
	γ-linolenate Biosynthesis II (Animals)	2.58	2.63 × 10^−3^	8.33 × 10^−2^	NaN	*ACSL1*, linoleic acid
***MSTRG.7472***	Acute Phase Response Signaling	1.12 x 10^1^	6.31 × 10^−12^	5.52 × 10^−2^	−0.378	*C5, FGG, HP, HPX, HRG, LBP, OSMR, SAA2, SOCS3, STAT3*
	Unfolded protein response	6.82	1.51 × 10^−7^	8.93 × 10^−2^	0.447	*CANX, DNAJC3, P4HB, PDIA6, XBP1*
	Role of JAK family kinases in IL-6-type Cytokine Signaling	4.64	2.29 × 10^−5^	1.20 × 10^−1^	NaN	*OSMR, SOCS3, STAT3*
	Role of JAK2 in Hormone-like Cytokine Signaling	4.24	5.75 × 10^−5^	8.82 × 10^−2^	NaN	*GHR, SOCS3, STAT3*
	Role of Tissue Factor in Cancer	3.85	1.41 × 10^−4^	3.36 × 10^−2^	NaN	*CFL1, FGG, P4HB, PDIA6*

NaN = not a number.

**Table 4:** Transcriptional upstream regulators for key lncRNAs with an activation score (except for *MSTRG.4802*: here all transcriptional regulators are listed).

Lnc RNA	Upstream Regulator	Activation z-Score	*p*-Value of Overlap	Target Molecules in Dataset
***MSTRG.4390***	PML	−2.433	1.22 × 10^−6^	*ACADM, APOA1, HADHB*, myristic acid, palmitic acid, stearic acid
	TP53	0.113	3.21 × 10^−2^	*ACADM, ACSL1, APOA1, HADHB, IDH1, INHBA, NDRG2, PCK1*
	SIRT1	0.317	8.98 × 10^−3^	*ACADM*, glycerol, *MAT2A, PCK1*
	MYC	0.577	2.51 × 10^−2^	*IDH1, INHBA, MAT2A, NDRG2, PCK1, SHMT2*
	SREBF1	0.652	1.69 × 10^−3^	*ACSL1, ARF4, IDH1, PCK1*
	HNF4A	1.181	8.09 × 10^−3^	*ACSL1, APOA1, HADHB, HSDL2, INHBA, MAT2A, MPP1, PCK1, RAB30, TRIP11*
	PPARGC1A	1.729	7.33 × 10^−5^	*ACADM, INHBA*, myristic acid, palmitic acid, *PCK1*, stearic acid
	PPARGC1B	2.177	4.51 × 10^−7^	*ACADM*, myristic acid, palmitic acid, *PCK1*, stearic acid
***MSTRG.4802***	PPARGC1B		3.03 × 10^−3^	*ATP5MF, COX5A*
	ARID5B		4.15 × 10^−3^	*UQCRB*
	Esrra		5.68 × 10^−3^	*ATP5MF, COX5A*
	PPARGC1A		6.22 × 10^−3^	*ATP5MF, ATP5PD, COX5A*
	HNF1A		1.44 × 10^−2^	*AP3M1, ATG3, CLTRN*
	KMT2D		1.85 × 10^−2^	*FBXL21P, PTGR2*
	SUB1		2.97 × 10^−2^	NDUFB10
	HTT		4.36 × 10^−2^	*AGRN, ATP5MF, UQCRB*
***MSTRG.5042***	PML	−2.000	1.89 × 10^−3^	*APOA1*, myristic acid, palmitic acid, stearic acid
	SREBF1	0.652	7.56 × 10^−3^	*ACSL1, ARF4, IDH1, PCK1*
	TCF7L2	0.728	2.99 × 10^−3^	*ACSL1, ADIPOR2, FBP1, IDH1, PCK1*
	HNF4A	1.505	1.03 × 10^−2^	*ACSL1, APOA1, ASGR2, FBP1, HSDL2, INHBA, MAT2A, PABPN1, PCK1, RAB30, RTCB, SOAT2, TRIP11*
	PPARGC1A	1.673	7.26 × 10^−4^	*GK, INHBA*, myristic acid, palmitic acid, *PCK1*, stearic acid
	SP1	1.934	2.66 × 10^−2^	*ACSL1, APOA1, MAT2A, PCK1, THRB*
	PPARGC1B	2.000	8.73 × 10^−5^	myristic acid, palmitic acid, *PCK1*, stearic acid
***MSTRG.7472***	STAT3	−0.877	6.51 × 10^−5^	*C5, FGG, HP, LBP, PDIA4, SOCS3, STAT3, XBP1*
	TP53	−0.640	3.11 × 10^−2^	*CD44, HDLBP, NARS1, P4HB, PDIA6, STAT3, TMSB10/TMSB4X, UGDH, XBP1*
	ATF4	−0.152	3.90 × 10^−5^	*CANX, NARS1, OSMR, SLC39A14, STAT3*
	CEBPB	−0.133	5.64 × 10^−5^	*HP, HPX, LBP, SAA2, SOCS3, STAT3, XBP1*
	NFE2L2	0.000	6.00 × 10^−8^	*C5, DNAJC3, GHR, NARS1, PDIA4, PDIA6, SOCS3, TMED2, UGDH, XBP1*
	XBP1	0.262	1.16 × 10^−6^	*DNAJC3, FKBP2, P4HB, PDIA4, PDIA6, SEC61G, XBP1*
	ATF6	0.762	1.50 × 10^−5^	*DNAJC3, PDIA4, SLC39A14, XBP1*
	TCF3	1.000	6.56 × 10^−8^	*AZGP1, EPRS1, GPLD1, NUF2, PDIA4, PDIA6, RASSF4, SOCS3, XBP1*
	TCF4	1.000	3.11 × 10^−4^	*NUF2, PDIA4, PDIA6, SOCS3, STAT3, XBP1*
	HNF1A	1.114	1.77 × 10^−6^	*C5, FGL1, HOPX, HPX, LBP, NUF2, SOCS3, TARS1, XBP1*
	PRDM1	1.176	1.91 × 10^−3^	*CD44, FGG, TRIB1, XBP1*
	HIF1A	1.932	3.21 × 10^−3^	*CD44, GHR, HP, SOCS3, STAT3*

**Table 5:** Phenotypic characteristics of bulls in high and low efficiency group.

Group	Number of Animals.	CF (%)		IMF (%)		RFI in MJ ME/day	
		Mean	SD	Mean	SD	Mean	SD
high efficiency	13	14.39	2.86	2.77	0.95	−20.91	4.47
low efficiency	13	20.28	4.06	4.59	1.71	20.48	4.40

CF = carcass fat content, IMF = intramuscular fat content in *M. longissimus dorsi*, RFI = residual feed intake, MJ ME = megajoule metabolizable energy, SD = standard deviation.

## Supplementary Materials

Supplementary materials can be found at https://www.mdpi.com/1422-0067/21/9/3292/s1.

## Abbreviations

lncRNA	long non-coding RNA
RFI	residual feed intake
QTL	quantitative trait locus
NAT	natural antisense transcript
FAANG	Functional Annotation of Animal Genomes
RIF	regulatory impact factor
PCIT	partial correlation and information theory
nt	nucleotide
FC	foldchange
PCA	principal component analysis
DE	differentially expressed
FPKM	fragments per kilobase million
IPA	Ingenuity Pathway Analysis
SD	standard deviation
CF	carcass fat
IMF	intramuscular fat content
Mb	megabase
