# Supplementary figures and images for: Identification and Annotation of Potential Function of Regulatory Antisense Long Non-Coding RNAs Related to Feed Efficiency in Bos taurus Bulls
Source: Int J Mol Sci. 2020 May 6;21(9):3292. doi: 10.3390/ijms21093292 (PMC7247587; doi:10.3390/ijms21093292)

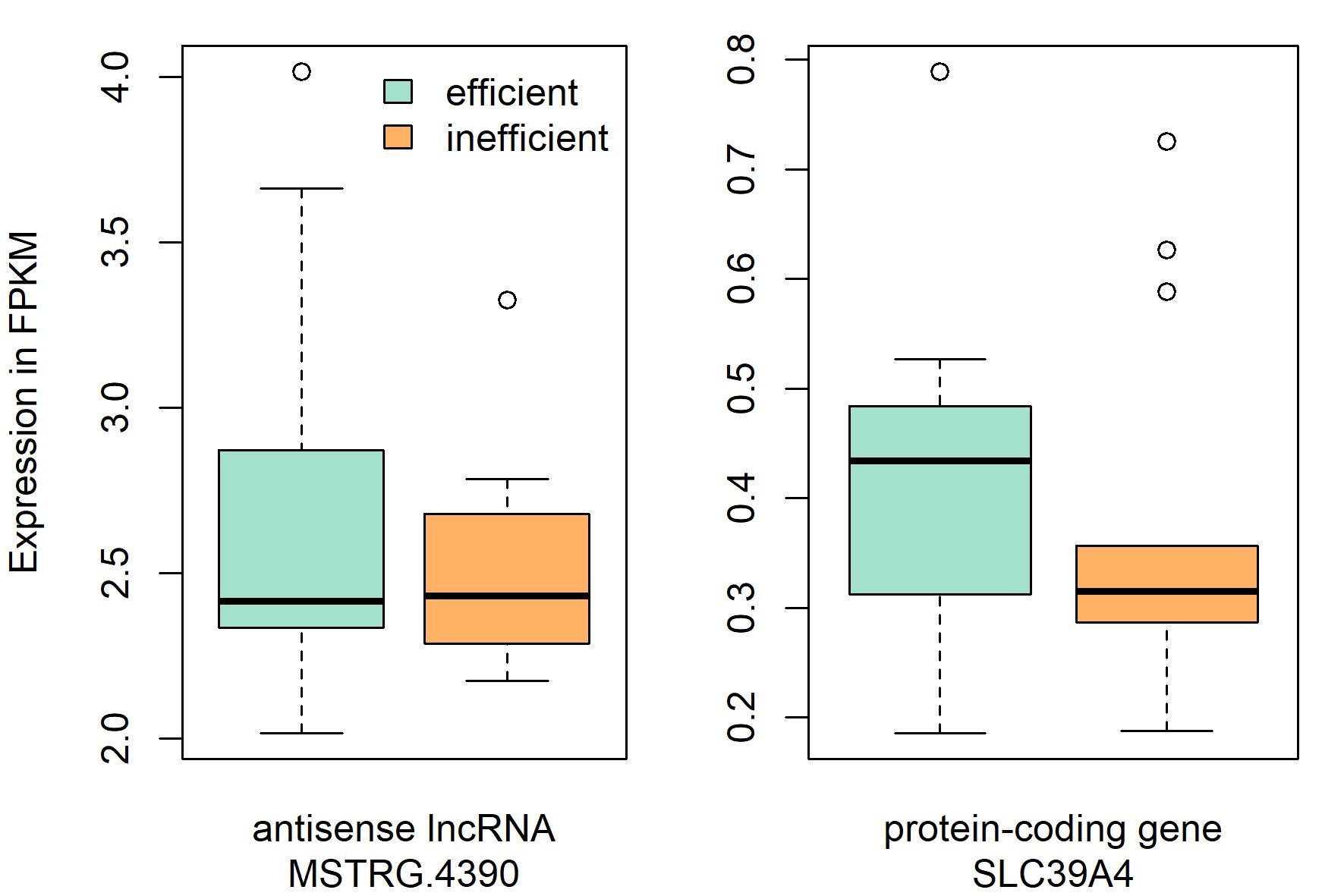

Supplement: Supplementary file 1 [file ijms-21-03292-s001.zip › Supplementary_Figure_1_MSTRG.4390_cis_partner_expression.jpeg]

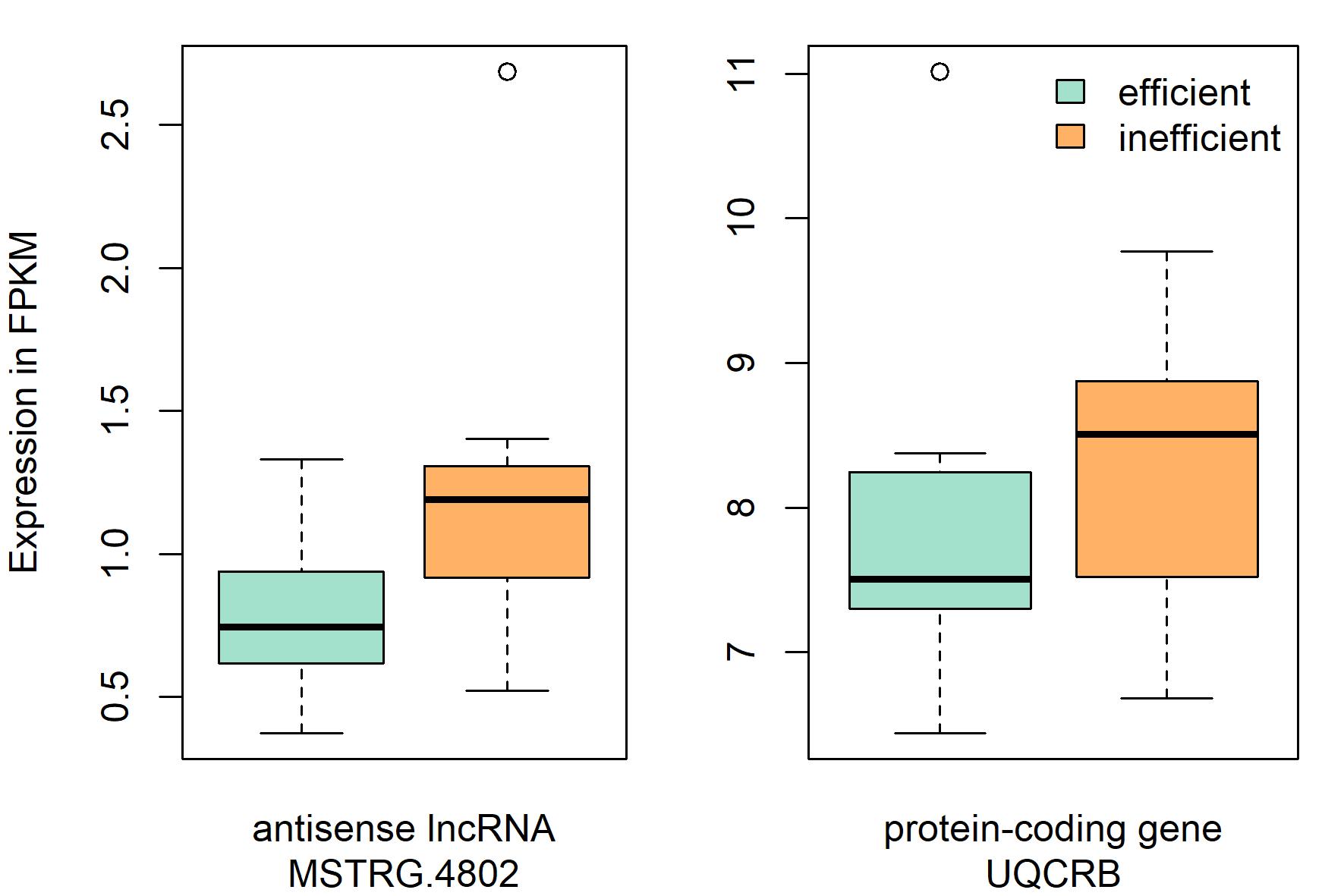

Supplement: Supplementary file 1 [file ijms-21-03292-s001.zip › Supplementary_Figure_2_MSTRG.4802_cis_partner_expression.jpeg]

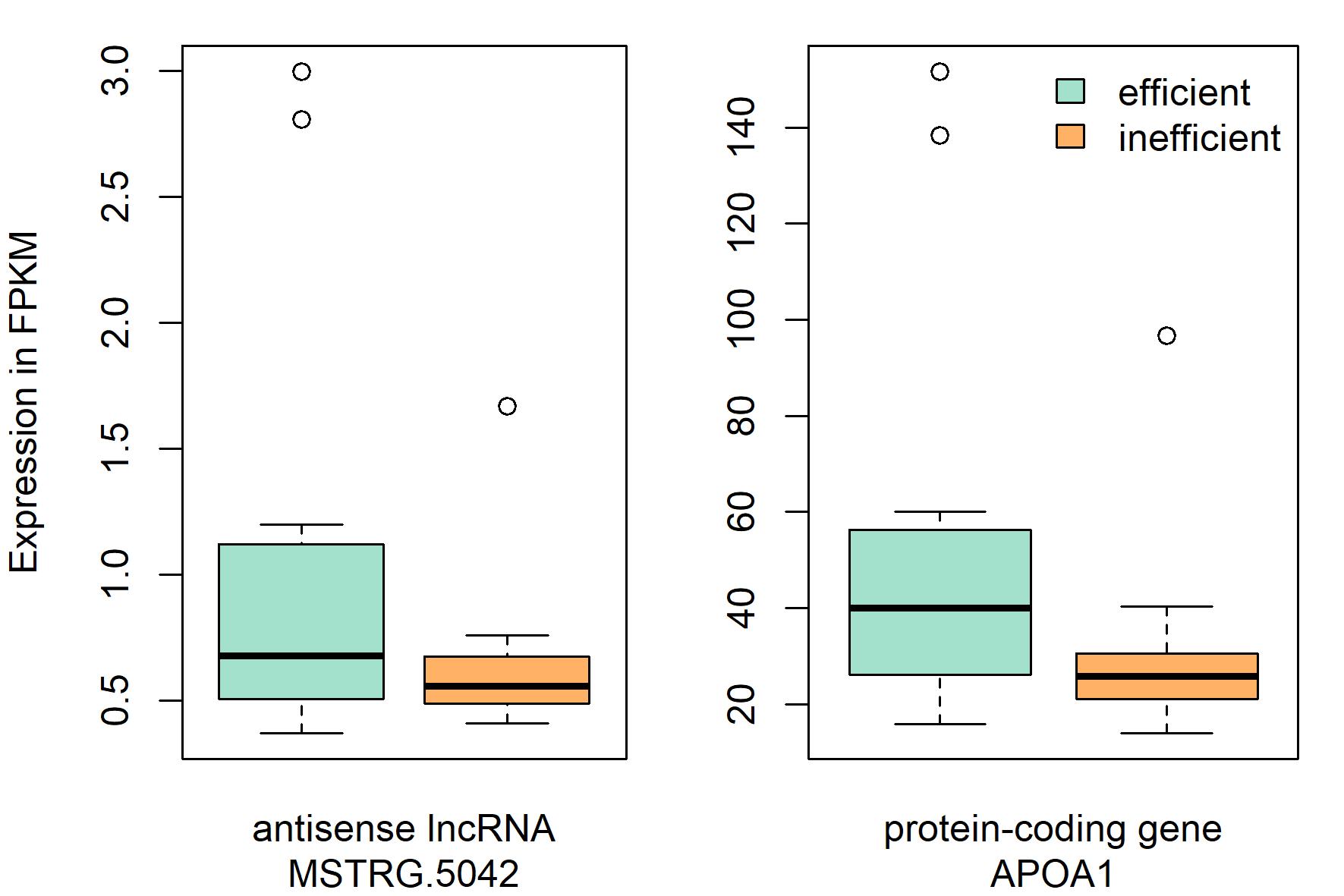

Supplement: Supplementary file 1 [file ijms-21-03292-s001.zip › Supplementary_Figure_3_MSTRG.5042_cis_partner_expression.jpeg]

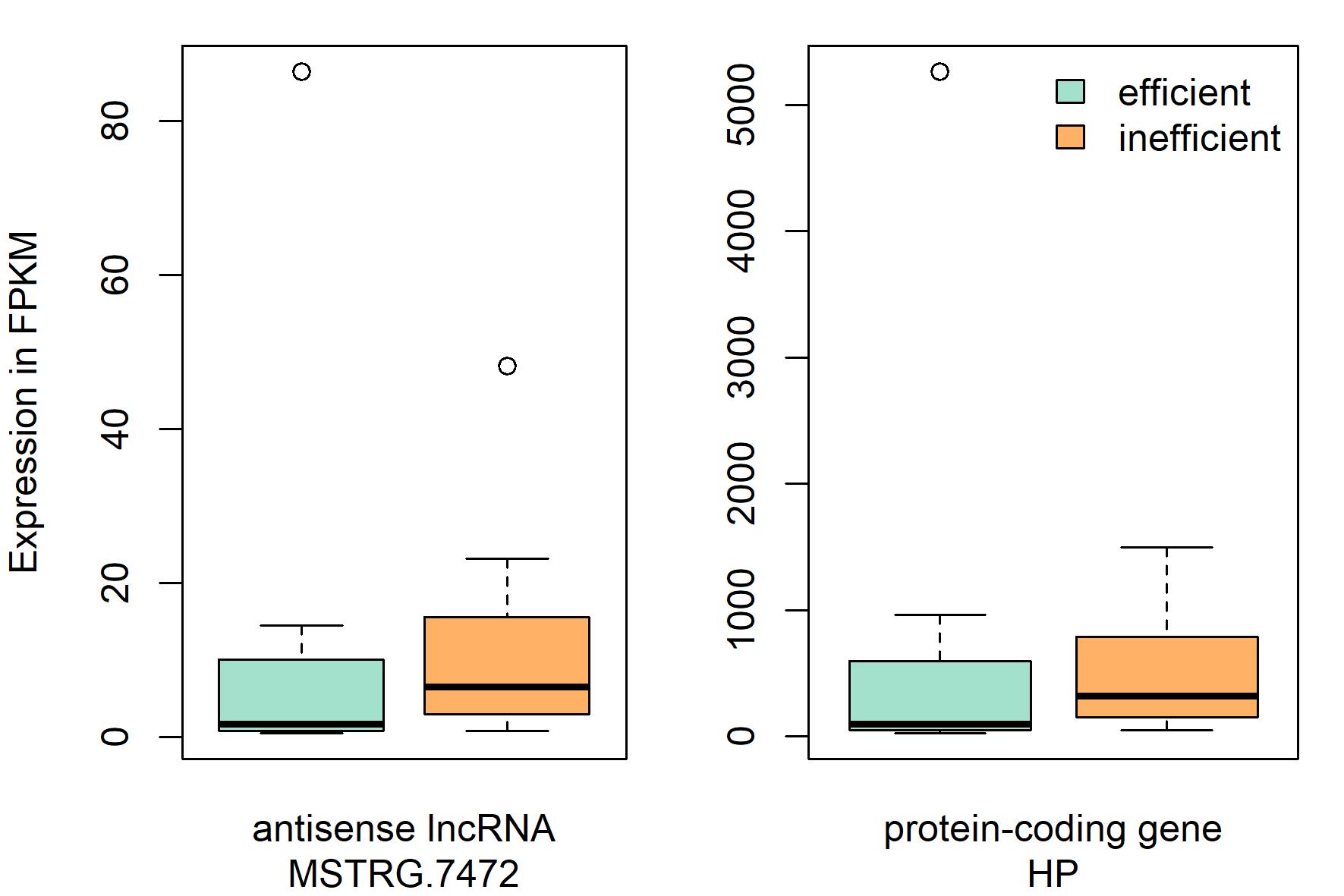

Supplement: Supplementary file 1 [file ijms-21-03292-s001.zip › Supplementary_Figure_4_MSTRG.7472_cis_partner_expression.jpeg]
